# Radiologic-pathologic analysis of increased ethanol localization and ablative extent achieved by ethyl cellulose

**DOI:** 10.1038/s41598-021-99985-4

**Published:** 2021-10-19

**Authors:** Erika Chelales, Robert Morhard, Corrine Nief, Brian Crouch, Jeffrey I. Everitt, Alan Alper Sag, Nirmala Ramanujam

**Affiliations:** 1 Department of Biomedical Engineering, Duke University, Durham, NC USA; 2 Department of Pathology, Duke University Medical Center, Durham, NC USA; 3 Division of Vascular and Interventional Radiology, Department of Radiology, Duke University Medical Center, Durham, NC USA

**Keywords:** Drug delivery, Biomedical engineering, Cancer therapy, X-ray tomography

## Abstract

Ethanol provides a rapid, low-cost ablative solution for liver tumors with a small technological footprint but suffers from uncontrolled diffusion in target tissue, limiting treatment precision and accuracy. Incorporating the gel-forming polymer ethyl cellulose to ethanol localizes the distribution. The purpose of this study was to establish a non-invasive methodology based on CT imaging to quantitatively determine the relationship between the delivery parameters of the EC-ethanol formulation, its distribution, and the corresponding necrotic volume. The relationship of radiodensity to ethanol concentration was characterized with water–ethanol surrogates. Ex vivo EC-ethanol ablations were performed to optimize the formulation (n = 6). In vivo ablations were performed to compare the optimal EC-ethanol formulation to pure ethanol (n = 6). Ablations were monitored with CT and ethanol distribution volume was quantified. Livers were removed, sectioned and stained with NADH-diaphorase to determine the ablative extent, and a detailed time-course histological study was performed to assess the wound healing process. CT imaging of ethanol–water surrogates demonstrated the ethanol concentration-radiodensity relationship is approximately linear. A concentration of 12% EC in ethanol created the largest distribution volume, more than eight-fold that of pure ethanol, ex vivo. In vivo, 12% EC-ethanol was superior to pure ethanol, yielding a distribution volume three-fold greater and an ablation zone six-fold greater than pure ethanol. Finally, a time course histological evaluation of the liver post-ablation with 12% EC-ethanol and pure ethanol revealed that while both induce coagulative necrosis and similar tissue responses at 1–4 weeks post-ablation, 12% EC-ethanol yielded a larger ablation zone. The current study demonstrates the suitability of CT imaging to determine distribution volume and concentration of ethanol in tissue. The distribution volume of EC-ethanol is nearly equivalent to the resultant necrotic volume and increases distribution and necrosis compared to pure ethanol.

## Introduction

Interventional radiology has the potential to provide care to a massive portion of the global population that does not have access to treatment for liver cancer, the fourth leading cause of cancer-related mortality globally as of 2018^1^. Although low- and middle-income countries (LMICs) will account for 70% of cancer mortality by 2040^2^, they receive only 5% of global cancer resources^3^. As a result, 9 out of 10 people in LMICs do not have access to basic surgical care^4^, transplantation rates are a tenth of those in high-income countries (HICs)^5^, and access to chemotherapy is often unreliable due to cost and insufficient testing infrastructure to direct treatment regimens^6^.

Thermal ablation is established as a curative treatment for hepatocellular carcinoma (HCC) in the Barcelona Clinic Liver Cancer staging criteria^7^. Compared to surgery it is less expensive^8^, less invasive^9^, faster^10^ and requires shorter hospital stays^11^. Ablation is well-tolerated and has gained widespread acceptance^12^ for treatment of primary tumors in the liver, kidney, and lung^13^, pre-cancerous lesions on the surface of the cervix^14^, metastases^15^, and chronic pain^16^. However, thermal ablation has a high barrier for entry to LMICs due to cost and the need for reliable electrical power. In contrast, ethanol ablation is inexpensive, easy to implement, and has been historically used to treat inoperable HCCs. Further, as a non-thermal modality, ethanol outperforms thermal ablation next to structures such as bowel, intestine, or gallbladder^17^, especially in a setting where there is no computed tomography (CT) access to allow reliable deep-field hydro-dissection. However, a caveat of using ethanol is leakage into neighboring tissue, which has resulted in unpredictable ablations, need for retreatment and off-target toxicity. For ethanol ablation to be an effective alternative to thermal ablation, ethanol must be localized within the target tissue^18^.

To achieve this goal in the treatment of venous malformations and herniated discs, ethanol is mixed with the water-insoluble polymer ethyl cellulose (EC) prior to injection^19,20^. The EC-ethanol mixture is a liquid, but upon injection into tissue and exposure to water, it undergoes a phase change into a cotton-like gel. Sequestration of the ethanol within the gel prevents it from leaking unpredictably from the injection site. Prior work from our group has demonstrated that EC-ethanol shrinks tumors in vivo and increases survival; a single infusion of EC-ethanol in a hamster model of oral squamous cell carcinoma increased tumor regression compared to pure ethanol^21^ and in a syngeneic model of breast cancer EC-ethanol decreased localized adverse events and increased overall survival^22^. Additionally, EC-ethanol ablation reduced tumor volume and was demonstrated as feasible in the treatment of felines with squamous cell carcinomas^23^.

Given the use of ethanol in the treatment of HCCs and the global incidence and mortality of the disease, our goal is to establish a quantitative and non-invasive strategy to determine the distribution of the injectate and determine its correspondence to the extent of resultant necrosis. To develop EC-ethanol ablation for clinical treatment of the liver, several factors must be optimized, including the formulation of the agent, the delivery of the agent, the injectate distribution and the relationship between the distribution and the extent of resultant tissue damage. In addition, an imaging approach is essential to quantify each of these steps, in vivo. Our prior work^24^, performed on excised swine liver, investigates the effect of the EC-ethanol formulation, needle insertion depth, infusion rate and volume on tissue fracture, infusion pressure and backflow, to minimize the off-target leakage of the agent. In this study, fluorescein was added to the injectate to allow for visualization of the distribution of ethanol after sectioning with a fluorescent microscope but this method is invasive, time consuming, and impractical for clinical use. Therefore, a non-invasive imaging strategy is required for these methodologies to be clinically translatable.

The current manuscript demonstrates a CT image guided strategy to assess ethanol concentration and distribution in tissue and establishes a quantitative relationship between the input parameters (delivery) to the output (extent of necrosis). This study was performed in both ex vivo and in vivo rat liver. We demonstrated that a concentration of 12% EC in ethanol creates the largest distribution volume and the lowest aspect ratio compared to that of 0, 6, 8, 10 or 15% EC-ethanol in ex vivo liver tissue. Further, 12% EC-ethanol resulted in an eightfold and threefold increase in ethanol distribution volume in ex vivo and in vivo livers, respectively, compared to that of pure ethanol. Radiological-pathologic correlation studies showed that 12% EC-ethanol yielded a necrotic zone sixfold greater than pure ethanol. Further, the average ratio of necrotic volume to ethanol distribution volume as observed with CT is greater for 12% EC-ethanol than for pure ethanol. Finally, a detailed time-course histological study was performed to assess tissue response to ablation including inflammatory and wound healing processes. The histological evaluation of the liver post-ablation with 12% EC-ethanol and pure ethanol revealed that while both induce coagulative necrosis and similar tissue responses at 1–4 weeks post-ablation, 12% EC-ethanol yielded a larger ablation zone.

## Results

### CT radiodensity values show a linear relationship with ethanol concentration

**Figure 1:**
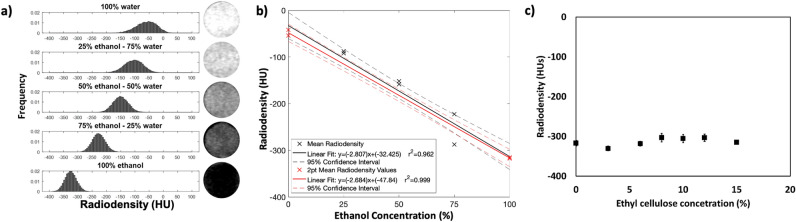
Measurement of ethanol concentration in water by computed tomography. (**a**) Radiodensity distribution and cross-sectional images of 0%, 25%, 50%, 75%, and 100% ethanol–water solutions demonstrate that ethanol has endogenous CT contrast. (**b**) Radiodensity values for ethanol–water solutions (n = 2 for each concentration) from a representative imaging session fit using a linear fit and a 2-point linear calibration equation with 95% confidence intervals. (**c**) Average radiodensity of EC-ethanol mixtures measured in triplicate. Error bars indicate standard deviation of average radiodensity value for a given EC concentration.

CT imaging is appropriate for distinguishing ethanol from tissue as ethanol attenuates X-rays less than water^25^. However, the use of this relationship to determine ethanol concentration in tissue has not been investigated. CT images of ethanol–water mixtures at 0%, 25%, 50%, 75% and 100% ethanol (n = 20 for each concentration) were acquired. Figure 1a shows representative histograms of the radio density distribution and corresponding cross-sectional CT images of the ethanol–water mixtures. The histograms show that the mean radiodensity decreases with increasing ethanol concentration (− 66.5 ± 20.3 HU for pure water versus − 340.3 ± 29.1 HU for pure ethanol (n = 20 for each concentration, p < 0.0001). The measured radiodensity is reflected by the scatter plot of a representative imaging session in Fig. 1b. A linear fit was applied to the mean radiodensities of each vial, yielding a correlation coefficient of ${r}^{2}$= 0.962. The data was also fit with a 2-point linear calibration equation using the radiodensities of 0% and 100% ethanol and yielded a correlation coefficient of 0.999. Both fits are shown in Fig. 1b with confidence intervals of 95%. While the ethanol concentration-radiodensity relationship is approximately linear, intermediate concentrations have a greater radiodensity than predicted by the 2-point linear calibration equation. The error introduced by this non-linearity, defined as the difference in ethanol concentration between the predicted and true values of the 25%, 50%, and 75% ethanol solutions, was 7.7% ± 6.8% (n = 20). The random component of the measurement error, or the variance in radiodensity of a sample of homogenous concentration, was also quantified. The variance is the average standard deviation of the radiodensity distribution of water–ethanol solutions totaling 20.8 ± 6.5 HU (n = 20), which is 7.6% ± 2.4% of the difference between the radiodensity values for ethanol and water. Therefore, we used the 2-point linear calibration equation to convert from units of radiodensity (HU) in CT images to estimated ethanol concentration and quantify the distribution of ethanol concentration in tissue.

To investigate whether the presence of EC affects the ethanol concentration estimation, EC was dissolved in ethanol at different concentrations and the mixtures were imaged by CT. Figure 1c demonstrates that radiodensity was not impacted by the presence of EC, verifying that CT imaging assesses the impact of EC on the spread of injected ethanol and not the presence of EC itself.

### 12% EC achieves the largest ethanol distribution volume and lowest aspect ratio of injected ethanol in ex vivo liver

**Figure 2:**
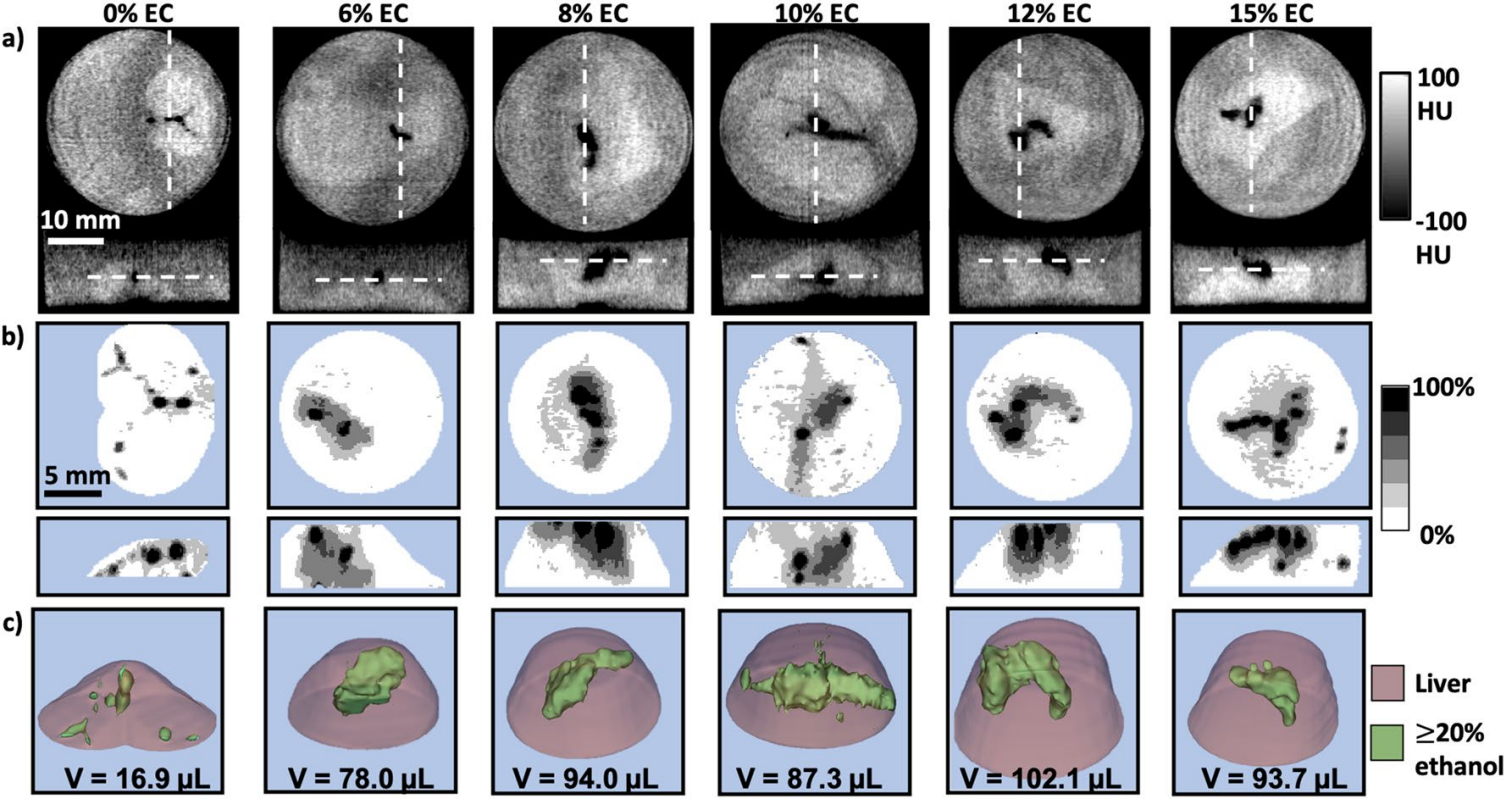
Visualizing the effect of EC concentration on injected ethanol distribution. (**a**) Representative transverse (top) and frontal (bottom) cross-sections of CT images acquired 5 min after injection of EC-ethanol into the ex vivo rat liver. White dashed lines represent the plane of the accompanying image. 100 µL of EC-ethanol at 0%, 6%, 8%, 10%, 12%, or 15% EC was injected at 10 mL/h. Tissue was submerged in buffer to prevent air absorption that would artificially lower the radiodensity. (**b**) Representative maximum concentration projections of transverse (top) and frontal (bottom) views, with the grayscale indicating 20% ethanol concentration bands. (**c**) 3D segmentations of ethanol distributions at different EC concentrations with the volume of each segmentation denoted below. Green regions contain at least 20% ethanol, and the pink region corresponds to segmented liver.

Clinical use of ethanol ablation has been limited due to leakage of ethanol from the injection site, reducing tumor coverage and causing adverse off-target effects^26^. Ethanol ablation can be improved by localizing the ethanol distribution within a defined region by using gel-forming polymers. We performed ablations in an ex vivo rat liver model to quantify ethanol localization following injection of EC-ethanol, defined by the volume and aspect ratio of the ethanol distribution within the tissue. Although previous work has demonstrated that incorporation of EC reduces leakage and improves efficacy of ethanol ablation^21^, the EC concentration which achieves a predictable, uniform distribution and reduces off-target leakage has not been established. EC-ethanol solutions containing over 15% EC have high viscosity and are difficult to inject with standard syringes and tubing; therefore, we capped the EC concentration at 15%. In each ex vivo rat liver sample, 100 µL of EC-ethanol^24^ at 0%, 6%, 8%, 10%, 12%, and 15% EC (n = 6 per group) was injected at a rate of 10 mL/h. Pre- and post-ablation CT images were acquired. Figure 2a shows representative transverse (top row) and frontal (bottom row) cross-sectional post-ablation CT images of containers of tissue submerged in buffer. Tissue appears brighter (higher radiodensity) than buffer; dark pixels (lower radiodensity) within the tissue correspond to injected ethanol. Maximum concentration projections, shown in Fig. 2b, display the 3D ethanol distributions maximally projected onto a 2D plane. Each maximum concentration projection was created by projecting the voxel with the highest ethanol concentration onto a 2D image in either the transverse (top row) or frontal (bottom row) perspective. Since the pre-ablation liver has naturally occurring regions of low radiodensity that appear similar to dilute ethanol, a minimum threshold of 20% ethanol ensures that these regions are not included in our analysis, as illustrated in Supplementary Fig. S5a. As in the images in Fig. 2a, the darker pixels of concentration projections in Fig. 2b correspond to higher ethanol concentrations. The images shown in Fig. 2b demonstrate that EC-ethanol ablation leads to regions of highly concentrated ethanol surrounded by regions of less concentrated ethanol within the tissue. Concentrations of 20% ethanol or higher are cytotoxic at the imaging time post-ablation^27^; therefore, the volume of ethanol within the tissue at an estimated concentration of 20% or greater was our primary metric for optimization. 3D segmentations of the volume containing an estimated concentration of 20% or greater, shown in Fig. 2c with the volume of each segmentation listed below, show the injected ethanol distributions (green) within the liver (pink).

**Figure 3:**
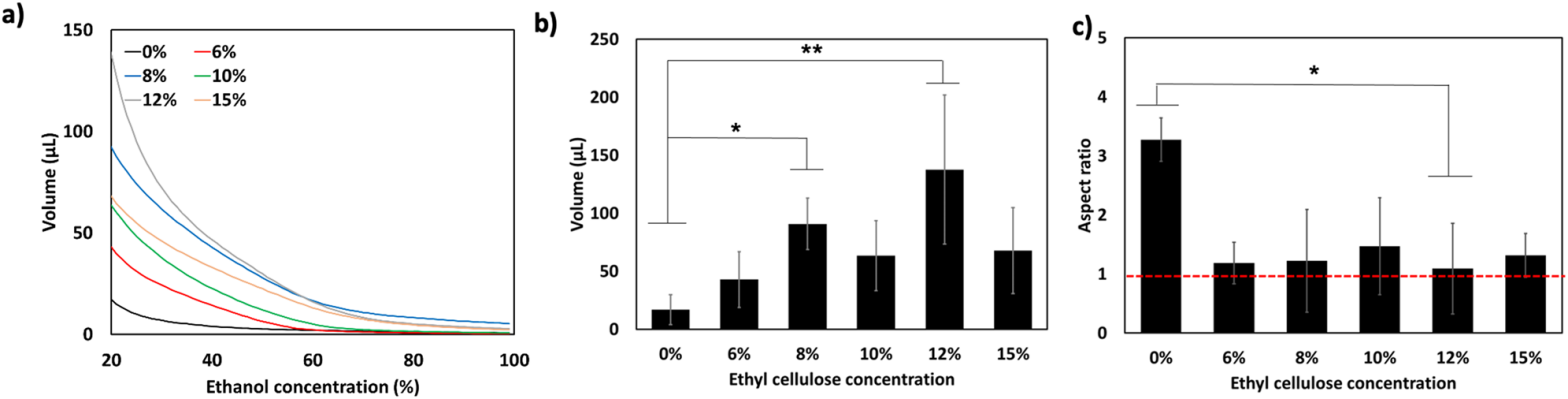
12% EC leads to the largest and most localized ethanol distribution. (**a**) Average cumulative volume histograms for each EC concentration. (**b**) Volume and (**c**) aspect ratio of the ethanol distribution. The red line denotes an aspect ratio of 1, Error bars indicate standard deviation. *p < 0.05 and **p < 0.01 (ANOVA and Tukey’s test) versus 0% EC-ethanol (n = 6 for each group).

Image data was converted from units of radiodensity to ethanol concentration with a linear two-point calibration equation (Eq. (1)) using pre-injection tissue radiodensity and the radiodensity of pure ethanol as the standard for 0% and 100% ethanol, respectively. Cumulative volume histograms (i.e. the average ethanol volume above a given concentration, n = 6), shown in Fig. 3a, illustrate that 12% EC in ethanol results in the largest distribution of ≥ 20% ethanol. Figure 3b shows the average pre- and post-ablation distribution volumes with estimated ethanol concentrations ≥ 20%. Pure ethanol had the lowest distribution volume, 17.1 ± 12.9 µL. The incorporation of 12% EC yielded the greatest distribution volume (137.7 ± 64.3 µL), which was significantly greater than that of ethanol alone (p < 0.01), an greater than eightfold improvement. Further, this distribution is closest to the actual injection volume of 100 µL. 12% EC-ethanol also yielded significantly greater distribution volumes than 6% (42.9 ± 24.8 µL, p < 0.01), 10% (63.5 ± 30.2 µL, p < 0.05), and 15% EC-ethanol (67.9 ± 37.0 µL, p < 0.05). The incorporation of EC not only increased ethanol accumulation within the tissue, but also led to a more localized ethanol distribution, quantified using the aspect ratio, as shown in Eq. (2), which is the ratio of the average distance of each point in the distribution to the centroid (radius of gyration) to the radius of a spherical distribution of equivalent volume (effective radius). A more localized distribution has a low aspect ratio, whereas more asymmetric distributions have greater aspect ratios. Localized distributions indicate minimal leakage and allow clinicians to plan the needle insertion location for lesions of any size; distributions with irregular shapes complicate ablation planning. Figure 3c shows the average aspect ratio for each injection group. Injections of 12% EC-ethanol produced the lowest aspect ratio (1.09 ± 0.12), significantly more localized than injections of pure ethanol (3.27 ± 2.83, p < 0.05).

### 12% EC-ethanol achieves a more localized injection distribution and greater volume of necrosis compared to conventional ethanol ablation in an in vivo liver model

**Figure 4:**
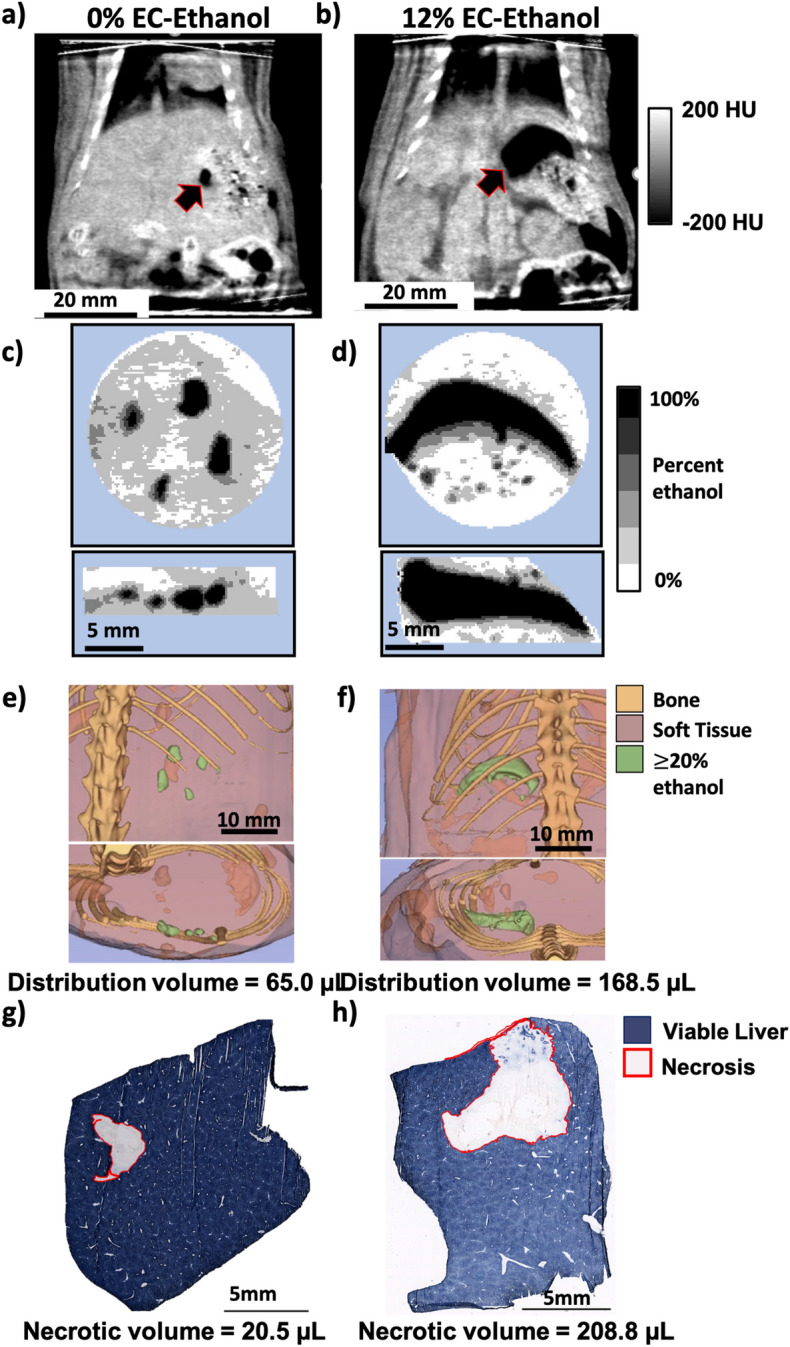
12% EC-ethanol improves localization and extent of ablation in rat liver in vivo. (**a**,**b**) Representative frontal plane CT images showing ethanol distribution (arrow) for 0% and 12% EC-ethanol. (**c**,**d**) Maximum intensity projections for the frontal (top) and transverse (bottom) views illustrate multiple foci of ethanol for 0% EC, and a single, large, connected distribution for 12% EC. (**e**,**f**) 3D segmentations from a frontal plane (top) and transverse plane (bottom) for injections of pure ethanol into in vivo rat liver depict small and unconnected ethanol distributions. In contrast, the 3D segmentations from a frontal plane (top) and transverse plane (bottom) for 12% EC-ethanol demonstrate a larger and completely connected ethanol distribution. The distribution volume for each sample is quantified under each representative image. (**g**) NADH-diaphorase viability staining of a representative section of tissue injected with pure ethanol shows a small area of necrosis (gray, outlined in red) surrounded by viable liver (blue). (**h**) NADH-diaphorase viability staining of a representative section of tissue injected with 12% EC-ethanol shows a larger area of necrosis (gray, outlined in red) surrounded by viable liver (blue). The volume of induced necrosis is quantified under each representative image.

12% EC in ethanol is most effective in maximizing the distribution volume and achieving an aspect ratio close to unity in an ex vivo rat liver model. Next, we investigated whether 12% EC-ethanol induced a larger zone of necrosis than pure ethanol in an in vivo rat liver model. Ablations were performed by injecting 100 µL of 12% EC-ethanol or pure ethanol (n = 6) at a rate of 10 mL/h into the left lateral liver lobe in vivo. CT images of the liver were acquired 10 min pre- and post-ablation. Tissue was excised 24 h post-ablation to determine the extent of ablation via pathologic analysis. Figure 4a,b depict frontal plane cross-sectional CT images of pure ethanol and 12% EC-ethanol ablations, respectively. Ethanol appears as a black area and is indicated by a black arrow within the liver. Figure 4c,d show maximum concentration projections, compiled using the same method as in Fig. 2b, for the frontal (top row) and transverse (bottom row) views for pure ethanol and 12% EC-ethanol ablations. For pure ethanol, the areas containing the highest estimated ethanol concentrations appear as multiple black foci, whereas the maximum concentration projections for 12% EC-ethanol depict a large continuous fluid distribution consisting mainly of concentrated ethanol. Figure 4e,f show segmented images from frontal and transverse views of a pure ethanol and a 12% EC-ethanol ablation, with the green region depicting the injected ethanol distribution at an estimated ethanol concentration of at least 20% and the pink region showing soft tissue. The pure ethanol distribution consists of multiple small, non-contiguous distributions. In contrast to pure ethanol, the segmentation for 12% EC-ethanol shows a single, contiguous ethanol distribution.

**Figure 5:**
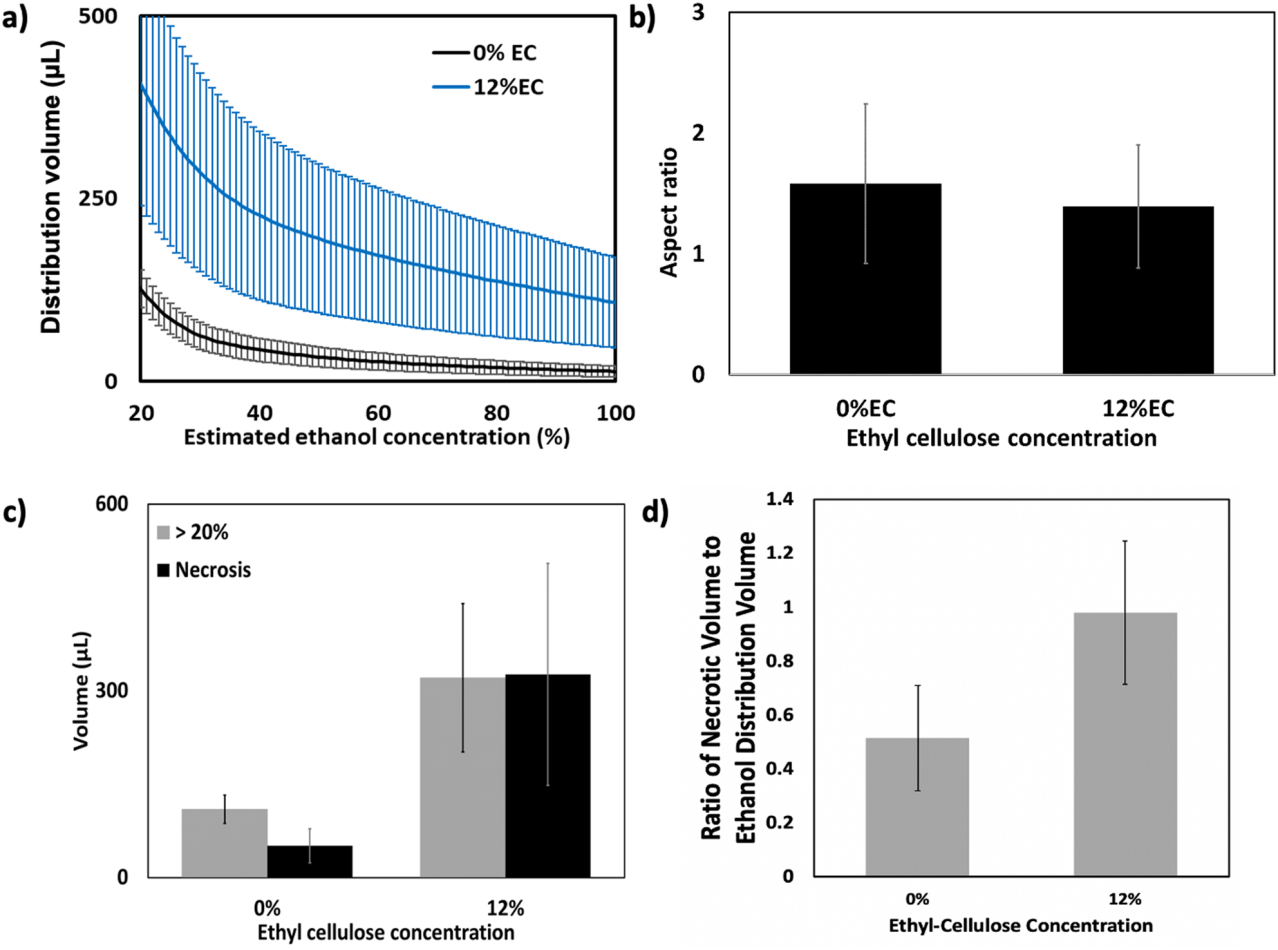
12% EC-ethanol improves localization of injected ethanol and effectiveness of ablation. (**a**) Average cumulative volume histograms for the injection distribution volume corresponding to the estimated ethanol concentration illustrate improved delivery for 12% EC-ethanol compared to pure ethanol. (**b**) The aspect ratio for pure ethanol is higher than that of 12% EC-ethanol indicating that distributions are less localized (N.S.) (**c**) Both the volume of induced necrosis and the ≥ 20% ethanol distribution volume are significantly greater for 12% EC-ethanol than for pure ethanol (p < 0.05, n = 6). (**d**) The average ratio of the necrotic volume to ethanol distribution volume is closer to 1 for 12% EC-ethanol than for pure ethanol (N.S.), indicating that CT imaging provides a more accurate prediction of necrosis for EC-ethanol than pure ethanol.

To determine the extent of ablation, tissue was excised 24 h after injection, cryopreserved, sectioned, and stained with NADH-diaphorase. NADH-diaphorase stains viable tissue blue and does not stain non-viable tissue. Representative sections of tissue stained with NADH-diaphorase after ablation with pure ethanol and 12% EC-ethanol are shown in Fig. 4g,h, respectively. The image shows a necrotic area, outlined in red, surrounded by viable liver tissue. To approximate the volume of induced necrosis, the area of necrosis on each slide was quantified by a process described in Supplementary Fig. S3 and verified with manual segmentation (Supplementary Fig. S4). To quantify volume, the necrotic area from each slide was multiplied by the sectioning step-size and summed. After ablation, animals did not exhibit mobility impairment, inflammation/edema, bleeding, respiratory distress, loss in body weight, hair coat changes, posture changes or lethality. The average cumulative volume histograms for pure ethanol and 12% EC-ethanol, shown in Fig. 5a, demonstrate that 12% EC-ethanol yields a larger distribution volume of ≥ 20% ethanol than pure ethanol. This analysis indicates that some tissue has an estimated ethanol concentration greater than 100%, possibly due to the inclusion of air bubbles within the tissue that may have entered the tissue during the open surgical procedure. The magnitude of this artifact in comparison to pure ethanol and the ex vivo injections is detailed in Supplementary Fig. S4. To account for the presence of air bubbles in our analysis, voxels with an estimated ethanol concentration > 120% were excluded in quantification of the ethanol distribution. Figure 5b illustrates that the aspect ratio for 12% EC-ethanol was also more favorable than that of pure ethanol (1.39 ± 0.51 vs. 1.58 ± 0.66, NS) indicating a more localized ethanol distribution.

Figure 5c shows in vivo ethanol distribution volumes and necrotic volumes for the ethanol and 12% EC-ethanol groups. Confirming our ex vivo results, an EC concentration of 12% led to a significantly greater distribution volume than pure ethanol (320.9 ± 291.9 µL vs. 109.6 ± 56.0 µL, p < 0.05), a near threefold improvement. The incorporation of 12% EC led to a > sixfold increase in necrotic volume versus pure ethanol (326.5 ± 436.4 µL vs. 50.9 ± 67.2 µL, p < 0.05). For each sample, the ratio of necrotic volume to ethanol distribution volume determined by CT was calculated and is shown in Fig. 5d. The average ratio of necrotic volume to ethanol distribution volume is greater for 12% EC-ethanol than for pure ethanol (0.98 ± 0.65 vs. 0.51 ± 0.48, NS).

### EC-ethanol and pure ethanol ablation result in coagulative necrosis

**Figure 6:**
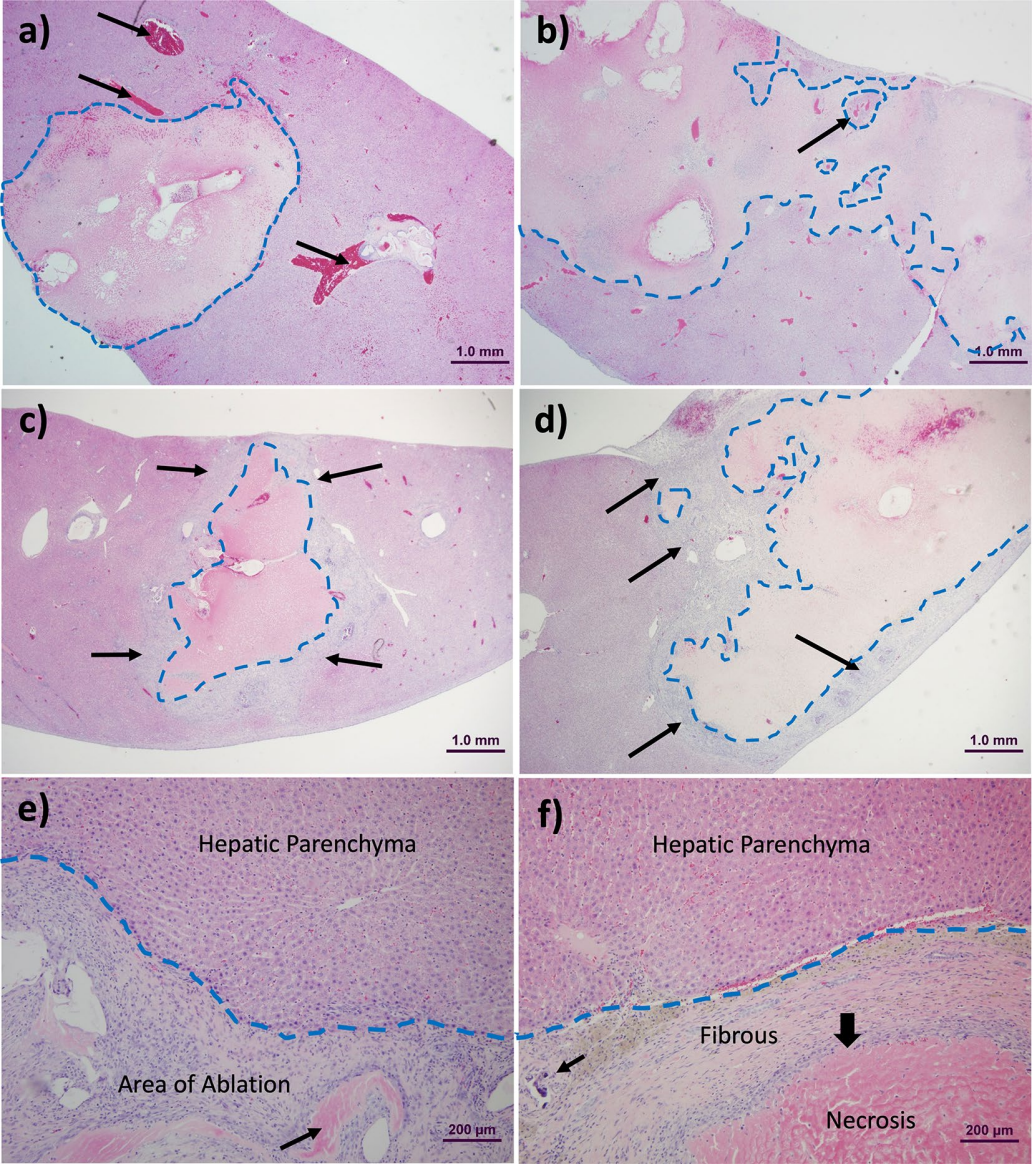
EC-ethanol and pure ethanol ablation cause coagulation necrosis with a similar wound healing response. (**a**) Photomicrograph of liver 72 h post-ablation with EC-ethanol with the border between coagulated necrosis and viable tissue denoted by the dotted blue line. Arrows indicate congestion in the acutely ablated hepatic parenchyma. (**b**) Hepatic tissue from liver 72 h post-ablation with pure ethanol with the outlined border between coagulative necrosis and viable tissue. Arrow depicts viable hepatocytes surrounding blood vessel. (**c**) Hepatic parenchyma of liver rat 1-week post-ablation with EC-ethanol. Note basophilic infiltrate of inflammatory cells (arrows) and granulation tissue walling off necrotic tissue from normal hepatic parenchyma. Dotted blue line indicates border of ablated zone. (**d**) Hepatic parenchyma of a liver 1-week post-ablation with pure ethanol (similar to **c**). Arrows depict proliferating and infiltrating cells at periphery of ablation zone. Dotted blue line indicates border of ablated zone. (**e**) Interface of ablation zone and viable liver 4-weeks post-ablation with EC-ethanol showing extensive cellular infiltrate of macrophages, inflammatory infiltrate, fibroblasts, and endothelial cells, interspersed with remains of necrotic tissue (arrow). Dotted blue line indicates border of ablated zone. (**f**) Interface of ablation zone and viable liver 4-weeks post-ablation with pure ethanol showing an area where there is extensive fibrosis and collagenization walling off viable hepatic parenchyma from a large island of coagulative necrosis in the ablation zone. Note the focus of mineralization (small arrow) and the infiltrate between the fibrous encapsulating tissue and the necrotic zone (thick arrow). Dotted blue line indicates border of ablated zone.

At 3 days post-ablation there was a prominent region of coagulative necrosis in the ablated liver for both EC-ethanol (Fig. 6a) and pure ethanol (Fig. 6b) groups. Pure ethanol ablation resulted in necrotic regions with more prominent islands of intact non-necrotic hepatocytes at the periphery of ablation zones at 3 days post-ablation (Fig. 6b). Although remains of hepatic architecture were noted in both groups, there was complete necrosis and loss of cytologic detail (e.g., nuclear structure) in all cells within the central portions of the ablation zone, including hepatocytes, sinusoidal lining cells, biliary structures, and vascular structures in portal regions. These changes including inflammatory cell infiltrates were present at 3 days post-ablation but particularly evident at the 1-week time point when acellular infiltrate completely circumscribed the tissue within the ablation zone (Fig. 6c,d). In peripheral areas of the ablated zone there was some preservation of vascular structures along with surrounding hepatic parenchymal cells (Fig. 6b (arrow), Supplementary Fig. S6a,b). Inflammatory cells and granulation tissue were prominent in the surrounding peripheral zone that demarcated normal from necrotic parenchyma (Supplementary Fig. S6c,d). At early time points in both treatment groups, marked congestion of the hepatic vasculature was present surrounding the zone of ablation.

Supplementary Fig. S6c and S6d demonstrate the cellular composition of the response one-week post-ablation, including inflammation and fibrosis demarcating the boundary between the ablated zone and viable tissue. Between 2- and 4-weeks post-ablation the ablated parenchyma became less cellular and more fibrotic with foci of mineralization found scattered in the zone of ablation along with increased collagenization walling off necrotic tissue (Fig. 6e,f). Histopathology of post-ablation livers at 3- and 4-weeks post-treatment showed healing of the hepatic parenchyma with diminution of areas of coagulative necrosis along with increasing amounts of granulation tissue, mineralization, fibrosis and hepatocytic regeneration than at the 3 day and 1 week time points (Supplementary Fig. S6e,f). Tissue reaction in the liver at 1 week through 4 weeks post-ablation was similar in both treatment groups, consisting of ingrowth of macrophages, lymphocytes, and fibroblasts as well as ingrowth of small vessels and bile duct hyperplasia as is associated with the regenerative and healing phases of coagulative hepatic necrosis (Supplementary Fig. S6 g,h).

The histopathology of ablation injury and repair in livers ablated with pure ethanol and EC-ethanol was similar over the 4-week time course of study with a single notable difference. In livers ablated with EC-ethanol there were small cystic areas containing transparent material believed to be EC with macrophages and multinucleate foreign body giant cells surrounding the material (Supplementary Fig. S6i). These granulomatous foci may be areas of removal of the EC polymer, but they are also undoubtedly the cellular reaction for the hepatic removal of cellular debris and necrosis of ablated tissue. Many macrophages and multinucleate giant cells were also present in livers ablated with pure ethanol without the presence of the transparent material (Supplementary Fig. S6j). While both 12% EC-ethanol and pure ethanol induce coagulative necrosis and similar tissue responses at 1–4 weeks post-ablation, only 12% EC-ethanol led to small cystic areas containing transparent material. Importantly, at each time point the hepatic parenchyma distal from the area of ablation appeared normal histologically.

## Discussion

The current study demonstrates suitability of CT imaging to define the delivery and uptake parameters of EC-ethanol in order to achieve near equivalence of the EC-ethanol distribution volume to the necrotic volume. The equivalency of these two volumes indicates that the distribution volume as determined by ethanol concentration extraction and concentration-based thresholding via CT imaging is an accurate representation of the resultant ablative effect. In contrast, the distributions visualized on CT for pure ethanol do not show equivalency to the resultant necrotic volumes, offering an explanation for why conventional ethanol ablation requires multiple treatment sessions or injections^18,30–32^. Although histopathology is the gold standard for demonstrating therapeutic effect, its use in the assessment of intratumoral drug delivery is challenging because tissue must be sectioned, and treated tissue often spans multiple sections. Aligning individual specimens to reconstruct the in vivo configuration is not possible because tissue preparation requires sectioning into multiple tissue blocks that deform during preparation. CT imaging is a quicker, non-destructive, and simpler method than histopathology to assess efficacy of ablation.

CT imaging provides a rapid (minutes), non-destructive, and three-dimensional method with an imaging depth of several centimeters and sub-microliter resolution which allows for high resolution imaging of injections in internal organs. CT imaging was a particularly effective tool for developing and establishing a non-invasive approach to quantify ethanol concentration in tissue, ethanol distribution volume and radial symmetry, and the relationship to the resultant necrosis. Ethanol concentration and distribution in tissue were imaged by CT and quantified using the natural contrast between ethanol and water^25^. Physical density is proportional to electron density, and therefore radiodensity^33^. Relative to water, ethanol has a lower density and fewer hydrogen bonds resulting in stronger X-ray absorption, and therefore lower radiodensity for ethanol^34^. Utilizing ethanol–water mixtures as in vitro tissue surrogates in this study, ethanol demonstrated a linear relationship between radiodensity and ethanol concentration^25^. For ethanol ablation to be effective, the target tissue must reach a cytotoxic concentration of 20% ethanol or greater^27^. Therefore, the linear relationship was fit with a 2-point linear calibration equation and used to segment a cytotoxic delivery threshold of 20% estimated ethanol concentration. This threshold also accounted for any small errors introduced by non-linearity and random noise. We found that ethanol alone has sufficient contrast with surrounding tissue therefore exogenous contrast agents were not used in this study.

Incorporation of ethyl cellulose (EC), an ethanol-soluble, water-insoluble polymer, induces formation of a fibrous gel phase upon exposure to tissue due to the high water content of tissue. The phase change of EC sequesters ethanol at the target site and achieves a more localized distribution comparative to pure ethanol. Liquid ethanol has low viscosity which allows for easier vascular clearance away from the target site, off-target leakage, and backflow, all of which limit ethanol accumulation and localization at the target site. A formulation of 12% EC-ethanol caused negligible leakage and the largest and most localized ethanol distribution volume in ex vivo rat liver, eight-fold greater than pure ethanol. This formulation also improved distribution volume by three-fold and increased the zone of pathologic necrosis by six-fold compared to pure ethanol in the in vivo rat liver. The phase change of the EC polymer allows for greater volumes of cytotoxic ethanol concentrations to accumulate compared to pure ethanol. Greater volumes of cytotoxic ethanol concentrations in tissue result in increased necrotic volume for EC-ethanol compared to pure ethanol. Further, EC-ethanol volume observed on CT was similar to the necrotic volume observed in histology, demonstrating reliable radiologic-pathologic correlation with CT as a non-invasive pathology surrogate. The comparative ratio of ethanol distribution-to-necrotic volume is also indicative of the ethanol release profile. The ratio of the ethanol distribution volume to necrotic volume was greater for 12% EC-ethanol than for pure ethanol, likely arising from the prolonged exposure time associated with gel formation similar to the delayed vascular clearance achieved by EC in the ablation of venous malformations^19^. Longitudinal imaging of the ethanol distribution is necessary to better understand this effect and confirmation of delayed clearance would make combination of EC-ethanol with other therapeutic agents synergistic as ethanol may extend the therapeutic effect.

Compared to pure ethanol, 12% EC-ethanol increases ethanol distribution volume more in the ex vivo studies than the in vivo studies, and the difference between the aspect ratios is significant for the ex vivo studies and not the in vivo studies. This disparity likely occurs because in the ex vivo samples the vasculature is collapsed and the tissue has been removed from the body and submerged in buffer. Therefore, the collapsed vasculature leads to a tissue-buffer barrier where the buffer provides little or no impedance to ethanol flow. In vivo the vasculature is not collapsed and contains flowing blood. Injections performed in vivo can be considered a closed system. Both pure ethanol and 12% EC-ethanol achieve greater distributions volumes in vivo compared to the volumes achieved ex vivo, suggesting that leakage out of the tissue into the buffer space decreases the observed volume. The comparative fold- increase is smaller in vivo since the closed system prevents leakage out of the tissue, which is more significant for pure ethanol, as it is less viscous.

Ethanol ablation induces coagulative necrosis by denaturing cellular proteins^35^ and dehydrating the cytoplasm^36^, and causes ischemia by damaging vascular endothelial cells^29^. We found that EC-ethanol achieves a larger and more well-defined ablation zone with fewer islands of non-necrotic hepatocytes than pure ethanol at three days post-ablation, but otherwise the histological responses of immune cell invasion and increased fibrosis at the ablation site at 1–4 weeks were similar for the two groups. Ablation lesions in livers in this study were similar to those previously reported in ethanol ablated rabbit^28^ and canine liver^29^. While histological studies cannot assess metabolism of the EC-ethanol, granulomatous foci visible in the liver ablated with EC-ethanol may indicate areas of removal of the ethyl cellulose polymer. The present study has limitations regarding determining the significance of residual EC in hepatic parenchyma. Future studies are necessary to investigate the role of EC in inflammation and residual granulomatous reaction. Previous studies demonstrate that gel mass decreases 79% after 24 h at 37 °C in vitro^21^ and previous clinical studies corroborate these results, showing spontaneous resorption of ethyl cellulose 3 weeks to 3 months after use in a sclerotic agent^19,37^. Further research is required to understand the mechanism of gel degradation and its affect wound healing in vivo.

We recognize several limitations of our study. First, we used a two-point calibration curve to calculate estimated ethanol concentration in ex vivo and in vivo experiments because creation of tissue-ethanol mixtures at known concentrations is not feasible. Although including intermediate points would increase accuracy, the error in estimated ethanol concentration is small compared to the volumes measured in the tissue samples and should not affect the conclusions drawn in our study. The distribution and efficacy of ablative therapies are commonly examined and optimized in both ex vivo and in vivo healthy tissue^38–46^, however, normal liver tissue is more homogenous than cancerous lesions. We plan to assess the impact of tissue heterogeneity on the ethanol distribution in cancerous lesions in future studies. Additionally, factors such as blood and bile flow and metabolic state may influence ethanol distribution in tissue. All animals were of the same age and species and allowed the same ad libitum access to water and food to control for significant differences in metabolic states. However, further metabolic markers or measurements of blood/bile flow rate were not acquired at the time of study. Animals were not fasted prior to imaging as this would lower liver radiodensity^47^, and reduce contrast when imaging the ethanol. Another limitation was the small size of the rat liver. The small size of the rat liver prevented analysis of larger infusion volumes which would be necessary to treat tumors clinically. Future studies will investigate larger infusion volumes and the potential need for multiple injections to ensure adequate tumor coverage. Further, both pure ethanol and 12% EC-ethanol showed a relatively large variance in the volume of ethanol accumulated in the tissue; however, it was difficult to ensure that the needle was placed in the center of the lobe and not near a large vessel, which would have artificially affected the distribution volume. Although not possible due to the small size of the animals in this study, clinically, ethanol ablations are often guided by ultrasound^32,35,48–53^ or CT^25,54^ to insert the needle to a precise location, mitigating issues associated with needle placement. For future large animal studies, the use of ultrasound or CT to guide needle placement and avoid large vasculature would likely reduce the variance in accumulated ethanol volume. Future studies may also investigate the effects of larger needle gauges or other needle types on ethanol distribution in tissue to inform clinical use. Finally, this study focused on the impact of EC concentration on ethanol distribution, but it has also been reported that longer EC chains result in stiffer gels. Further, the ethoxyl content of the EC dictates solubility and gel formation^55^. However, this study did not vary the ethoxyl content of the EC. Future work could investigate the effect of EC chain length and ethoxyl content on the extent of gel formation and flow of injected ethanol by constructing an ethyl cellulose-ethanol–water phase diagram^56^. Future studies could also quantify the impact of EC chain length and ethoxyl content on the ethanol concentration profile and injection distribution in real-time with the methods described in this study.

This optimized form of ethanol ablation is poised for immediate application in LMICs and HICs alike. The improved delivery achieved here obviates the need for large ethanol volumes and multiple treatment sessions limiting adverse non-target effects. While thermal ablation is the first-line therapy for liver tumors^57^ in HICs, it is not well-suited for tumors proximal to vital structures such as bile ducts, intestinal loops, or the gall bladder, or along the capsule^17^ because heat is more readily transmitted between organs than liquid. As a result, ethanol ablation is often recommended for these high-risk locations^58^ and EC incorporation should improve targeting. In LMICs, EC-ethanol is appealing because it eliminates the loss to follow-up, does not require multiple sessions^59^ as would be required with conventional ethanol ablation and is poised to expand access as it is low-cost, minimally invasive, highly portable, and electricity independent. The growing accessibility of ultrasound^60^ and its widespread history in targeting ethanol ablation^61^ facilitates image-guided ablation of primary tumors or metastases in the kidney, liver, and breast with either curative or palliative intent when no alternate treatment is available. Ultrasound is a particularly attractive alternative for use in settings where CT is unavailable, and the methodologies used here can be translated to ultrasound imaging in future work. Further, EC-ethanol ablation could supplement cryotherapy and thermocoagulation to treat superficial lesions such as oral and cervical precancerous lesions without the need for hard-to-supply consumables^62^. With growing interest in injectable stimuli-response polymers^63^ to locally deliver chemo- and immunotherapy^64,65^, assessment of intratumoral delivery is crucial to implementation. These studies characterized CT imaging for use in future optimization of EC-ethanol ablation protocols and established a methodology that can be implemented in the development of intratumoral delivery of other therapeutic agents. Our methodologies can be used to assess delivery and concentration of other therapeutic agents quantitatively and non-invasively in vivo, specifically to optimize the delivery, distribution, and the intended therapeutic outcome.

## Methods

### CT imaging acquisition and segmentation

All CT images acquired were full rotation (360°) with 180 projections at a 50 ms settlement time, medium magnification (pixel size, 78.81 µm), and a field of view of 8.07 × 16.11 cm, in a 512 × 512 matrix. Images were acquired at 80 kV, 500 µA with 300 ms exposure time. For in vivo images, a preset beam-hardening correction was applied. Images were acquired of ethanol-water vials and ex vivo and in vivo rat livers. Images were processed in 3D Slicer^66^. The average radiodensity of in vitro ethanol-water samples was computed by segmenting a cylinder within each vial. Ex vivo liver images were segmented by selecting the tissue surrounding the injected ethanol distribution without including surrounding buffer. Ethanol was segmented by interpolating between circles of 5–15 mm on each side of the injected ethanol. Overlapping circles were used when necessary. In vivo images were segmented by selecting the tissue surrounding the injected ethanol without including surrounding tissues (intestines, stomach, or lungs). The same method used for ex vivo images was used to segment the in vivo liver and ethanol distributions. Sample pre- and post-ablation segmentation volumes were similar. Segmentation and cross-sectional images were generated directly from 3D Slicer, analysis of segmentations and generation of histograms was done in MATLAB, and results were plotted in Microsoft Excel.

### Determination of ethanol concentration and quantitation of error from radiodensity measurements of ethanol vials

Ethanol–water solutions at 0%, 25%, 50%, 75%, and 100% ethanol (n = 20 each) were prepared to assess ethanol CT contrast. Fluorescein-ethanol solutions at 0%, 0.25%, 1%, and 2.5% fluorescein (n = 3 each) were also imaged as this formulation was used in our prior studies to visualize ethanol distribution in frozen tissue cross-sections^24^. Fluorescein was included in the ex vivo studies in case of CT malfunction requiring manual sectioning and distribution segmentation as reported previously^24^. Samples containing up to 2.5% fluorescein had the same radiodensity of ethanol (data not shown). Solutions examined are detailed in Table 1. 200-proof ethanol (anhydrous ethanol, Koptec, King of Prussia, PA) was mixed with water, EC (Sigma Aldrich, 247,499, St. Louis, MO), or fluorescein (Sigma Aldrich, F2456) at room temperature in a sealed container within 24 h preceding imaging. EC is linear chain of cellulose rings with a fraction of the hydroxyl groups replaced with ethoxyl groups (48–49.5% for this source). The average molecular weight for this source has been characterized by high-performance liquid chromatography as 80.8 ± 24 kDa with a polydispersity index of 102.5 ± 10.5^67^.

**Table 1:** Solutions of 200-proof ethanol mixed with water, ethyl cellulose (EC), or fluorescein (Sigma Aldrich, F2456) and imaged with CT.

Solution	Concentrations tested (%)
Ethanol–water	0, 25, 50, 75, 100 (n = 20)
Ethyl cellulose-ethanol	0, 3, 6, 8, 10, 12, 15 (n = 3)
Fluorescein-ethanol	0, 0,25, 1, 2.5 (n = 3)

Solutions were imaged with CT and segmentation was performed as described above. Radiodensity data was converted to ethanol concentration with a linear two-point calibration equation (Eq. (1)). Calibration was performed at the beginning of each imaging session. For in vitro experiments, vials of pure ethanol and water were imaged to serve as 100% and 0% ethanol standards, respectively. In the liver studies, the 0% ethanol standard was the average radiodensity of the pre-ablation liver since tissue has slightly higher radiodensity than water. The radiodensity difference of the 0% and 100% standards represents the radiodensity range for all possible ethanol concentrations (denominator). The radiodensity difference between the sample and the 0% ethanol standard was calculated (numerator) and divided by the radiodensity range for all possible ethanol concentrations (denominator) to determine the sample ethanol concentration. Equation (1) assumes a linear relationship between ethanol concentration and radiodensity, and that sample thickness does not affect measurement accuracy. Supplementary Fig. S1 illustrates application of this equation.

1 $${\text{Ethanol concentration}} = \frac{{{\text{Radiodensity}}_{{{\text{sample}}}} - {\text{Radiodensity}}_{{0\% {\text{ ethanol standard}}}} }}{{{\text{Radiodensity}}_{{100\%{\text{ ethanol standard}}}} - {\text{Radiodensity}}_{{0\%{\text{ ethanol standard}}}} }} \times 100\%$$

### Description of animal work

All animal studies were approved by the Duke University Institutional Animal Care and Use Committee and performed in accordance with guidelines and regulations (Protocol Number A160-18–07). Studies were performed in compliance with the ARRIVE guidelines. Male Fischer CDF rats (Charles River Laboratories) were used for ex vivo (n = 36 lobes, n = 10 rats), in vivo studies (n = 12 rats), and histological time course studies (n = 19). Male rats were used since anatomical imaging and ethanol concentrations should be insensitive to gender and liver cancer incidence is higher in males than females^1^. Rats had ad libitum food and water access and regular 12-h light/dark cycles. Studies were performed in healthy rat liver to allow for direct comparison of the EC-ethanol and pure ethanol distribution and ablative effect. Tumors are heterogeneous in cellular and microenvironment composition, but also in shape and size, while healthy liver tissue is relatively homogenous from sample to sample, providing a controlled environment to develop our methodologies, which can then be extended and tested in tumor studies. Further, the distribution and efficacy of ablative therapies are commonly examined and optimized healthy liver tissue^38–46^.

### Ex vivo rat liver studies

Rats were euthanized via isoflurane overdose and bilateral thoracotomy. The liver was immediately excised and stored in Krebs–Ringer bicarbonate buffer (Sigma Aldrich, K4002) on ice until injection (within 1–2 h). Individual lobes were placed in a small plastic container (height, 62.6 mm; diameter, 41.9 mm) for injection. A 27-gauge needle was lowered to the approximate center of the lobe using a holder to prevent lateral motion. We previously found that smaller 31G needles led to high pressures within the needle when injecting increasingly viscous solutions (3 or 6% EC-ethanol), and therefore required high forces applied to be applied to the syringe^24^. Since high forces on the syringe limit future applications in which fluid is manually injected, 27G needles were a preferred choice. Fluid was infused from a 3-mL syringe (BD Medical, Columbus, NE) through 10 cm of rubber tubing (1/4″ inner diameter, McMaster-Carr, Douglasville, GA) using a syringe pump (NE-1000, New Era, Farmingdale, NY) at a flow rate of 10 mL/h. Prior work demonstrated that 10 mL/h is optimal compared to 0.1, 1, and 100 mL/h^21,24^. 100 µL of fluid was infused based on infusion volumes previously optimized to reduce leakage^24^ and the small size of the rat liver. The needle was removed 3 min after infusion to allow fluid to dissipate. Non-contrast CT images of the samples were acquired pre- and post-injection with EC-ethanol (0%, 6%, 8%, 10%, 12% or 15%).

### In vivo rat liver studies

Pre-ablation non-contrast CT images of the rat abdomen were acquired. Rats were maintained with 1.5% isoflurane at 2 L/min during the procedure and a heating pad maintained body temperature. Buprenorphine Sustained-Release (1 mg/kg) was administered subcutaneously as an analgesic. The abdomen was depilated and disinfected three times with 10% povidone-iodine followed by 70% ethanol. A laparotomy was performed by creating an incision with a sterile scalpel through the skin and abdominal wall to expose the left lateral lobe of the liver. A sterile cotton-tipped applicator was used to expose the center of the left lateral liver lobe. Injections were performed as described above for the ex vivo studies. The needle was slowly retracted, and a cotton-tipped applicator was used to stop any visible bleeding. The abdominal wall was closed with Reli monofilament sutures (VWR, 89219-212). 1–2 drops of 0.25% bupivacaine were applied along the incision as a local anesthetic. The skin was closed with Coated VICRYL® (polyglactin 910) sutures (VWR, 95057-014). Post-injection non-contrast CT images of the liver were acquired.

Animals were monitored post-ablation every 6–8 h for 24 h for: mobility impairment; inflammation/edema; bleeding; respiratory distress; loss in body weight; licking, biting, scratching or shaking of procedure site; hair coat changes; posture; and lethality. Rats were euthanized by isoflurane overdose 24 h after ablation and the liver was immediately excised. The left lateral lobe was cut into three 2 × 2 cm samples. Samples were placed into Peel-A-Way^®^ disposable embedding molds (Polysciences Inc., 18646A-1, Warrington PA), labeled, and covered in optimum cutting temperature (OCT) gel (Sakura Finetek, Torrance, CA). The molds were placed in a metal container of 2-methylbutane (Sigma, 277258) and frozen using liquid nitrogen. Samples were stored in a − 80 °C freezer.

### Time course histological studies

Rats (210–285 g) were randomly assigned to treatment with either pure ethanol or 12% EC-ethanol and received ablations via a laparotomy procedure as described above. One to two rats from each treatment group were then euthanized 3 days, 1 week, 2 weeks, 3 weeks, and 4 weeks post-ablation. Rats were euthanized by isoflurane overdose and the liver was immediately excised. Hepatic tissues from the injection area of ablation were fixed in 10% neutral buffered formalin, processed to paraffin, sectioned at 5 microns, and stained with hematoxylin and eosin. The resulting slides were evaluated by an experienced board-certified veterinary pathologist with experience in toxicologic pathology (JIE). Sections were evaluated in a masked fashion with respect to treatment group to determine if differences were detected in tissue response, including inflammatory and wound healing responses, bile duct and blood vessel damage, and cellular reactions.

### Ethanol distribution volume and radial symmetry of ex vivo and in vivo liver tissues

Maximum intensity projection images were produced from the 3D segmentation by projecting the voxel with the highest estimated ethanol concentration onto a 2D image from top- and side-view perspectives. Projection mages were generated using custom-made MATLAB code that is available upon request. A 20% ethanol concentration threshold was used because a 10-min exposure of 20% ethanol is cytotoxic^27^ and images were acquired 10–20 min after injection. Supplementary Fig. S2 illustrates that this threshold excludes regions of naturally low radiodensity in untreated tissue from the analysis. Ethanol distribution volume was defined by the volume of tissue with ethanol concentration $\ge$ 20%. The degree of asymmetry of the ethanol distribution was quantified by the aspect ratio, as in Eq. (2)—the radius of gyration over the effective radius—for all voxels with estimated ethanol concentration $\ge$ 20%.

2 $$Aspect\;ratio = \frac{{Radius\;of\;gyration}}{{Effective\;radius}} = \frac{{\sum {Distan ce\;from\;centroid/number\;of\;pixels} }}{{\sqrt[3]{{3*Volume/4\pi }}}}$$

### Pathologic evaluation of ablative extent

To assess the extent of necrosis, two 7-µm sections were cut serially every 1 mm from frozen samples at − 15 °C with a cryostat microtome (Microm HM 560, Thermo Fisher Scientific, Waltham, MA). Serial sections were adhered to positively-charged, uncoated glass slides (Thermo Fisher Scientific, 6776214). One slide from each pair was stained with reduced nicotinamide adenine dinucleotide (NADH)-diaphorase, a viability stain which distinguishes viable cells (blue) from necrotic cells (unstained). The slides were covered in Tris buffer (0.05 M, pH 7.6) with 8 mg/5 mL NADH (Sigma, N8129) and 10 mg/5 mL nitro blue tetrazolium (Sigma, N6876) and incubated for 15 min at 37 °C. Slides were then washed three times with deionized water followed by three exchanges in 30%, 60%, and 90% acetone. The slide was covered in 90% acetone until a purple cloud appeared in the solution. Slides were washed three times with deionized water and allowed to dry. Coverslips (Thermo Fisher Scientific, 12540C) were applied with aqueous mounting medium prepared by mixing 21 mL of deionized water, 4 g of store-grade unflavored gelatin, 25 mL of glycerol (Sigma, G2025), and 0.5 mL of phenol (Sigma, P9346) at low heat. Sectioning was performed beginning with the sample from the distal end of the liver lobe. The second sample was sectioned and stained using the same procedure. If no necrosis was observed in any sections in the second sample, the third sample was not sectioned or stained; otherwise, the third section was sectioned and stained using the same procedure.

### Image analysis of pathology specimens from in vivo liver tissues

Slides were digitally scanned at 10 × magnification with a Zeiss Axio Imager Z2 upright microscope. The region of interest (ROI), or necrotic area, was determined using a custom MATLAB program. Images were cropped to remove portions that did not contain tissue. First the tissue was segmented by applying an entropy filter to the blue channel of the images and binarizing the result using a user-defined threshold. Small regions (< 15,000 connected pixels) were deleted to remove noise. The edges of the regions were eroded using a flat structuring element with a 15-pixel neighborhood, holes in the regions were filled, and the edges of the regions were dilated using the same structuring element. The boundaries of the tissue sample were detected, and the area was quantified using the MATLAB function ‘regionprops’. A mask generated from the binary image was used to remove background pixels from the original image.

The resultant image was then used to segment the necrotic area. The blue channel was binarized with a user-defined threshold, and small regions < 5000 pixels were removed. All ROIs except for the five largest were deleted from the image. Regions such as large vasculature which may be detected under the same threshold used for necrosis were manually selected for removal. The boundaries of the necrotic regions were detected and the MATLAB function ‘regionprops’ was used to quantify area. Supplementary Fig. S3 shows representative images each step. Necrotic volume was calculated by multiplying necrotic area by the sectioning step size (1 mm) and taking the sum for all samples for each animal. All images were processed by one user. Adjacent H&E slides were used for confirmation. The semi-automated MATLAB algorithm was compared to gold standard manual segmentation using ImageJ software. Digital images from the sections of two samples (1 per group) were manually segmented. For the 13 images assessed, the MATLAB algorithm estimated an average of 0.0465 cm^2^ more necrotic area than manual segmentation, with an average absolute scalar difference of 0.0049 cm^2^. Supplementary Fig. S4 shows the comparative analysis of the necrotic areas determined manually and using the MATLAB algorithm.

## Supplementary Information

Supplementary Information.
