# Supplementary material for: Radiologic-pathologic analysis of increased ethanol localization and ablative extent achieved by ethyl cellulose
Source: Sci Rep. 2021 Oct 19;11:20700. doi: 10.1038/s41598-021-99985-4 (PMC8526742; doi:10.1038/s41598-021-99985-4)
Supplement: Supplementary file 1 — Supplementary Information. [file 41598_2021_99985_MOESM1_ESM.docx]

# Radiologic-pathologic analysis of increased ethanol localization and ablative extent achieved by ethyl cellulose

# Erika Chelales ^1*^, Robert Morhard ^1*^, Corrine Nief^1^, Brian Crouch^1^, Jeffrey I. Everitt^2^, Alan Alper Sag^3^, and Nirmala Ramanujam^1^

1Department of Biomedical Engineering, Duke University, Durham, NC, USA

2Department of Pathology, Duke University Medical Center, Durham, NC, USA

3Department of Radiology, Division of Vascular and Interventional Radiology, Duke University Medical Center, Durham, NC, USA

*Denotes co-first authors

Corresponding author: Erika Chelales (erika.chelales@duke.edu)

Supplementary Figures
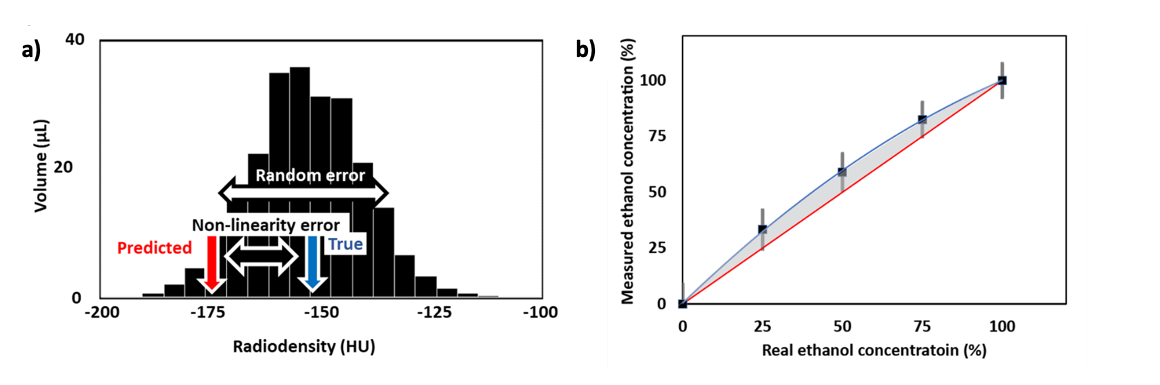


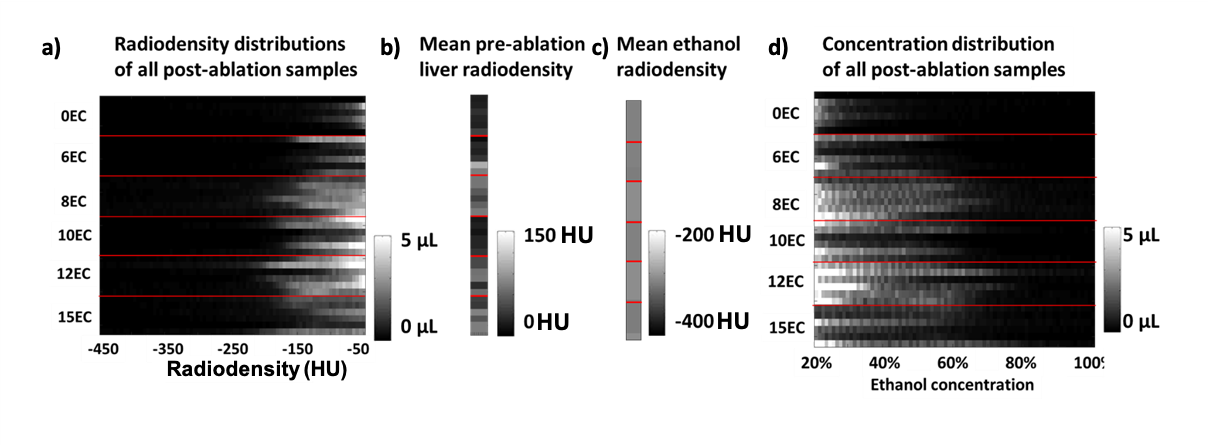
Supplementary Figure S1. a) Representative histogram of a 50% ethanol-50% water mixture with the magnitude of random error depicted by the white arrow. The magnitude of the non-linearity error is depicted by the black arrow as the difference between true (blue arrow) and predicted (red arrow) radiodensity. b) Average radiodensity values for ethanol-water solutions (n=20 per group) depict the relationship between ethanol concentration and radiodensity, fit by a two-point normalization equation (1) calculated from pure ethanol and pure water samples shown in red to generate predicted values. The non-linearity error is quantified as the difference between the predicted (red line) and true (blue line) values and is represented by the gray shaded region. Error bars indicate the average standard deviation of the radiodensity distribution for a given ethanol concentration.

Supplementary Figure S2. a) Individual grayscale histograms depict the radiodensity distribution for each sample. The darkness of each bin (width, 1 HU) corresponds to the volume of tissue at a given estimated ethanol concentration. Lighter bins correspond to a larger volume. b) Mean pre-ablation radiodensity values for each sample; lighter bands correspond to higher radiodensities. c) Mean ethanol radiodensities for each treatment group shown with a grayscale, with lighter bands indicating higher radiodensities. d) Grayscale histograms depict the estimation of ethanol concentration distribution for each sample. The darkness of each bin (width, 1% estimated ethanol concentration) corresponds to the volume of tissue at a given estimated ethanol concentration. Lighter bins correspond to a larger volume.


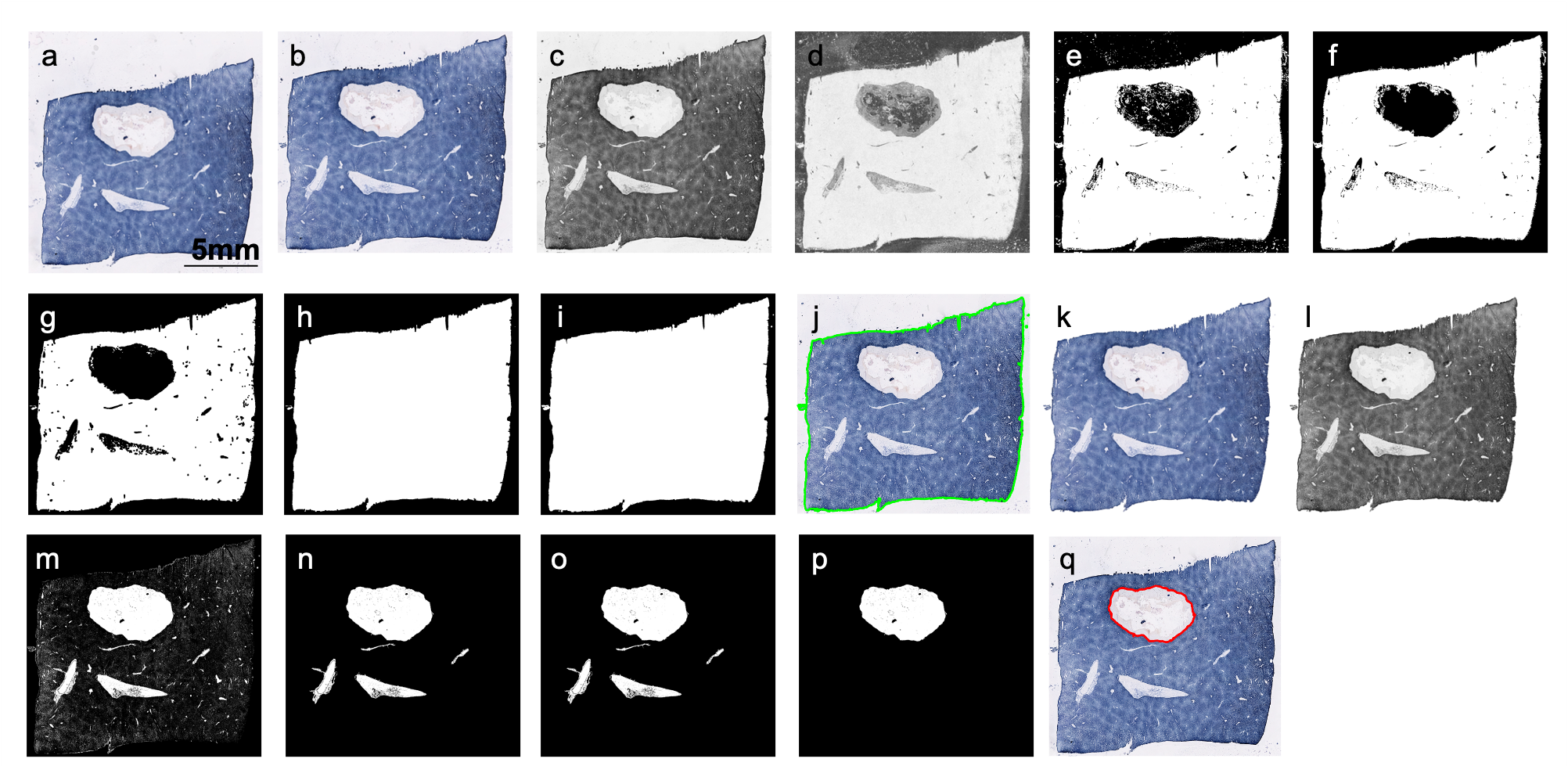


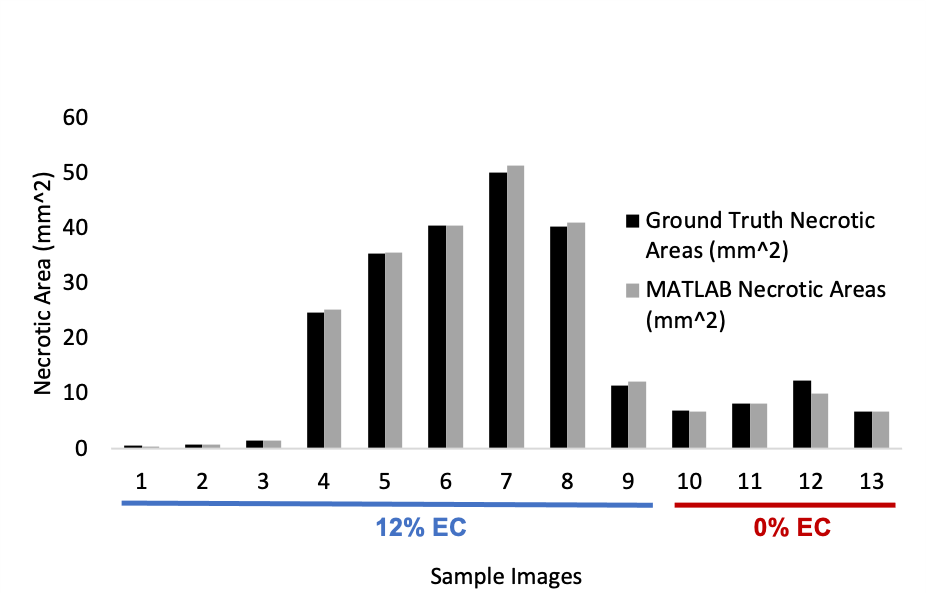
Supplementary Figure S3. Detection of necrotic area in a single image of tissue stained with NADH-diaphorase by a semi-automated MATLAB algorithm. Image sequence: a) original image, b) cropped image, c) blue channel, d) entropy filter, e) binary image, f) remove noise, g) erode, h) fill holes, i) dilate, j) detected sample, k) background removed, l) blue channel, m) binary image, n) remove noise, o) remove smaller ROIs, p) deselect large vasculature, q) detected necrosis.

Supplementary Figure S4. Two tissue samples, one treated with 12% EC-ethanol and one with pure ethanol (0% EC), were segmented manually in ImageJ as a ground truth for comparison to segmentation with a MATLAB algorithm. A total of 13 images were segmented between the two samples. On average, MATLAB estimated 0.0465 cm^2^ more than manual segmentation, with an average absolute scalar difference in necrotic area of 0.0049 cm^2^.


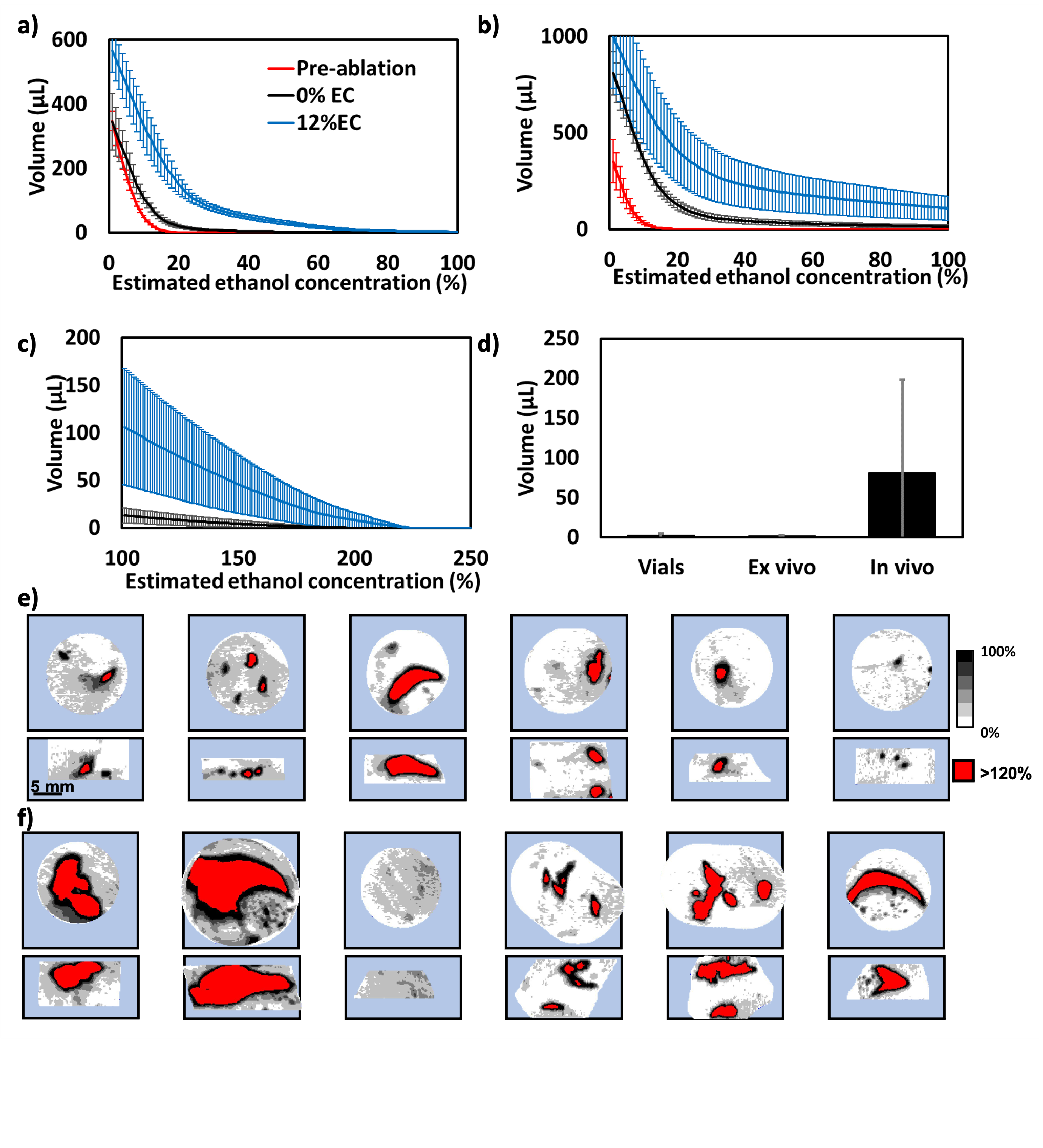


Supplementary Figure S5. a-b) Average CDFs of estimated ethanol concentration for all pre-ablation liver samples, and post-ablation 0% and 12% EC-ethanol samples *ex vivo* (a) and *in vivo* (b). c) Average CDFs of estimated ethanol concentration for estimated ethanol concentrations greater than 100% ethanol for all *in vivo* samples. d) Volume in the vials, *ex vivo* samples, and *in vivo* samples with an estimated ethanol concentration >120%. e-f) Maximum intensity projection images for all pure ethanol (e) and 12% EC-ethanol (f) *in vivo* ablations. Ethanol concentration is indicated by the grayscale; air is red.

Supplementary Figure S6. High magnification photomicrographs of features of the histological response to ablation. a-b) Photomicrographs depicting viable hepatocytes (arrow) surrounding portal vessel at periphery of ablation zone 3 days post-ablation with pure ethanol (a) and EC-ethanol (b). c-d) Arrows depict circumscribing band of inflammation and fibrosis that surrounds necrotic hepatic tissue 1-week post-ablation with pure ethanol (c) and EC-ethanol (d). e-f) Low magnification photomicrographs show the appearance of the ablation zone at 3 weeks post-ablation with pure ethanol (e) and EC-ethanol (f). Asterisks mark cystic zones surrounded by necrotic hepatic tissue and arrows indicate a circumscribing thick band of fibrosis and inflammatory infiltrate. g-h) Bile duct proliferation (arrows) at periphery of ablation zone in liver 1-week post-ablation with pure ethanol (g) and EC-ethanol (h). i) Photomicrograph of multinucleate foreign body giant cells (arrows) in the peripheral zone of liver 1-week post-ablation with pure ethanol. j) Photomicrograph of multinucleate foreign body giant cells (arrows) surrounding cystic space (asterisk) filled with transparent material believed to be EC at 1-week post-ablation with EC-ethanol.
